# A general differential split-sample test to select sub-periods of discontinuous years gathering similar to different climate conditions

**DOI:** 10.1016/j.mex.2020.101008

**Published:** 2020-07-23

**Authors:** Hamouda Dakhlaoui, Denis Ruelland, Yves Tramblay

**Affiliations:** a LMHE, Ecole Nationale d'Ingénieurs de Tunis, University of Tunis El Manar, BP 37, 1002 Tunis le Belvedère, Tunisia; b Ecole Nationale d'Architecture et d'Urbanisme, University of Carthage, Rue El Quods, 2026 Sidi Bou Said, Tunisia; c HydroSciences Montpellier (Univ. Montpellier, CNRS, IRD), Montpellier, France

**Keywords:** Rainfall-runoff modeling, Split-sample tests, Parameter transferability, Semi-arid climate

## Abstract

This article introduces a Matlab© code to implement the General Differential Split Sample Test (GDSST) (Dakhlaoui et al. [5]). As an illustration, the GDSST is applied to five catchments in northern Tunisia over 30-year reference period and compared to three benchmark Split Sample Test (SST) methods. The techniques are compared as regards to the number of validation exercises and to the differences in temperature (ΔT) and precipitation (ΔP) between the sampled sub-periods, whose length was set to 8-year. The GDSST allows a larger number of discontinuous periods to be sampled, and is computationally more effective than the basic bootstrap to identify the most climatically contrasting conditions. In addition, the GDSST offers a larger continuum of climatic conditions and a better spread of validation periods than the benchmark techniques, which is essential to test the parameter transferability of hydrological models. As supplementary material, a package file containing MATLAB© scripts to run the three benchmark SSTs and the proposed GDSST, as well as an application example on the five catchments, can be freely downloaded.

• An enhanced split-sample test based on an oriented bootstrap to assess transferability of hydrological models.
• The proposed split-sample test is computationally more effective than the basic bootstrap to identify the most climatically contrasting conditions.
• MATLAB© code of the proposed GDSST and four benchmark SST, with application example.

## Graphical abstract

**Figure:**
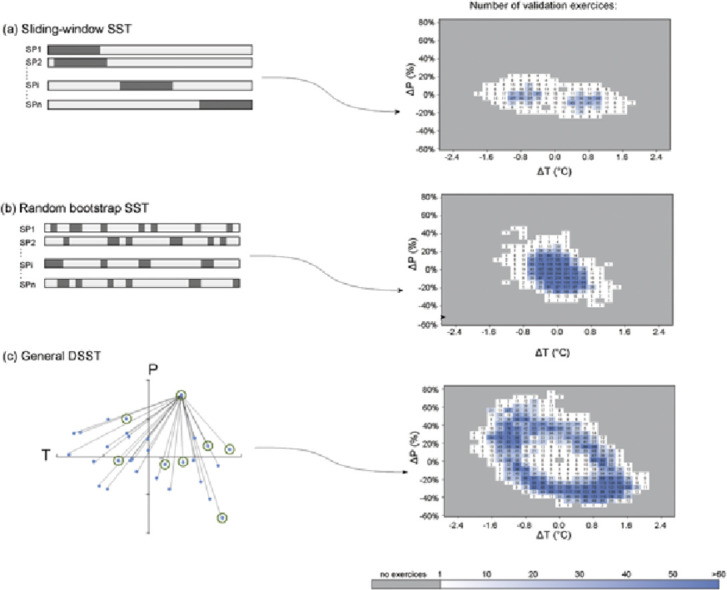

Specifications Table

**Table:** 

Subject Area	Earth and Planetary Sciences
More specific subject area	*Hydrology* *Hydroinformatic*
Method name	*General differential split-sample test (GDSST)*
Name and reference of original method	*Coron, L., Andréassian, V., Perrin, C., Lerat, J., Vaze, J., Bourqui, M., Hendrickx, F., 2012. Crash testing hydrological models in contrasted climate conditions: an experiment on 216 Australian catchments. Water Resour. Res., 48, W05552. doi:*10.1029/2011WR011721 *Coron, L., 2013. Les modèles hydrologiques conceptuels sont-ils robustes face à un climat en évolution ? PhD Thesis, ISIVE, AgroParisTech, 364 p.* *Dakhlaoui, H., Ruelland, D., Tramblay, Y., Bargaoui, Z., 2017. Evaluating robustness of conceptual rainfall-runoff models under climate variability in northern Tunisia. J. Hydrol., 550, 201‒217. doi:*10.1016/j.jhydrol.2017.04.032 *Dakhlaoui, H., Ruelland, D., and Tramblay Y. (2019). A bootstrap-based differential split-sample test to assess the transferability of conceptual rainfall-runoff models under past and future climate variability. Journal of Hydrology.*https://doi.org/10.1016/j.jhydrol.2019.05.056*.*

## Introduction

This article introduces a transferable package (see supplementary material) of the General Differential Split Sample Test (GDSST) to select sub-periods of discontinuous years gathering similar to different conditions in terms of precipitation and temperature. The GDSST was originally proposed in Dakhlaoui et al. [5] to assess the transferability of conceptual rainfall-runoff models under past and future climate variability. In this paper, we showed that compared to three existing benchmark techniques, the GDSST allowed a larger number of climatically contrasted discontinuous periods to be sampled, and was computationally more effective than the basic bootstrap to identify the most contrasted periods. When applied to three hydrological models in five catchments in northern Tunisia, the GDSST provided clear transferability limits of the models under changing precipitation (*P*) and temperature (*T*) conditions towards drier and hotter conditions. We also showed that some climate projections of temperature and precipitation from the EURO-CORDEX exercise fell outside these transferability limits.

Since a specific research method was customized for the above article, we thought readers might be interested in accessing the codes developed to run the proposed GDSST, the three benchmark techniques, as well as the application example on the five studied catchments. The current paper thus focuses on this technical part of our work as a description of a MATLAB package.

## Description of the split-sample techniques included in the package

### Three benchmark SST techniques

**Fig. 1:**
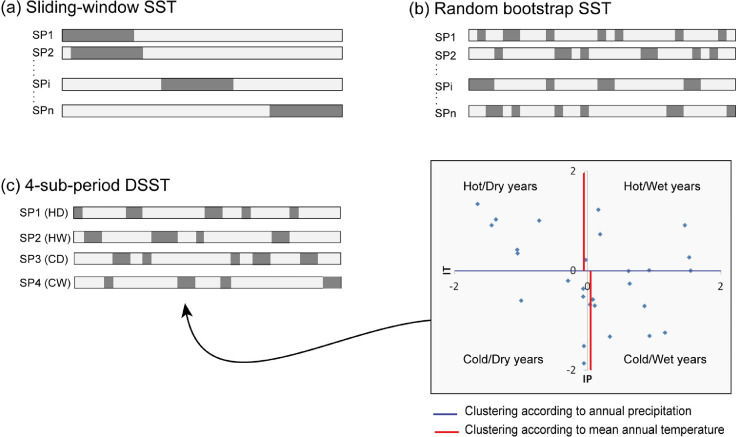
Split-sample methods according to (a) a sliding-window SST technique, (b) a random bootstrap SST technique, and (c) a 4-sub-period DSST technique [5].

The SST methods included in the package and selected for comparison with the proposed GDSST are (Fig. 1): (i) a sliding-window SST [2]; (ii) a random bootstrap SST [1,3]; and (iii) a 4-sub-period DSST [4]. These three techniques were selected because they enable simultaneous investigation of the effect of T and P on model transferability under climate variability.

The sliding-window SST technique [2] consists in using calibration-validation tests on independent sub-periods of equal length, considering all possible pairs of sub-periods. The sampling method used to generate sub-periods is based on sliding windows applied over the reference period. The technique enables the identification of *n*-*l* + 1 calibration sub-periods, where *l* is the number of years composing each sub-periods and *n* is the number of years of the reference period.

The random bootstrap SST technique [1,3] relies on a sub-period sampling technique which is based on a random combination of discontinuous years (bootstrap). This sampling technique is time consuming since the possible number of calibration sub-periods is equal to $C_{n}^{l}$. For example, the random bootstrap SST technique results in around six million possible 8-year sub-periods if applied to a 30-year reference period. Its application then requires a priori selection of the number of permitted calibration exercises, due to limited time budget for model calibration and validation.

The implementation of the 4-sub-period DSST [4] requires the calculation of the annual precipitation and mean temperature for each hydrological year of the reference period. The sub-periods are thus made up of groups of climatically contrasted years. To create these groups, the hydrological years are first distributed into two equal groups of hydrological years (dry years and wet years) according to the annual precipitation median for the reference period (Fig. 1c). Dry and wet years are defined as years with respectively less or more total precipitation than the median of the reference period. For each group, the median of the mean annual temperature is then calculated, which serves to distinguish hot and cold years. The four final groups of hydrological years are: hot/dry (HD), hot/wet (HW), cold/dry (CD) and cold/wet (CW) years (Fig. 1)

Using the three above techniques makes it possible to identify different numbers of calibration sub-periods of *n* years. All *n*-year periods which do not have any year in common with a given *n*-year calibration period can thus be considered as independent validation exercises. As a result, the number of validation exercises may not be the same for all calibration periods selected with the sliding-window and random bootstrap SST. For the 4-sub-period SST, there are three possible validation exercises for each of the 4 calibration sub-period.

### Proposed general differential split-sample test (GDSST)

**Fig. 2:**
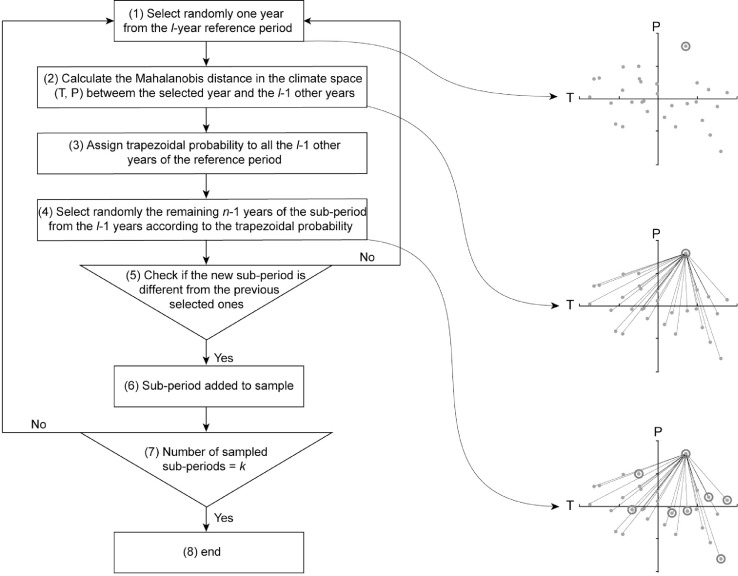
Processing steps to sample *k* climate contrasted l-year sub-periods from n-year reference period, in the proposed GDSST. Each point represents a hydrological year from the reference period in the climate space (*T, P*) . The years circled are those selected [5].

Based on the existing SST methods, we developed a technique which can take benefit from the random bootstrap SST technique to provide a large number of validation exercises while accounting for the much contrasted *ΔT* and *ΔP* detected with the 4-sub-period DSST. In other words, the idea was to design a method which uses the sampling of the random bootstrap SST technique, but which is oriented so as to obtain the extreme climate contrast provided by the 4-sub-period DSST. The proposed method was called general differential split-sample test (GDSST) and is described in Fig. 2.

The procedure used to generate *k n*-year sub-periods from the *l* hydrological years (from the 1st of September to the 31st of August) of reference period, is as follows. The first year of the *n*-year sub-period to be sampled is randomly selected from the *l* years of the reference period (step 1 in Fig. 2). The *l*-1 remaining years of the reference period are then sorted based on the order of increasing distance of Mahalanobis [6] to the first selected year in the space of mean annual temperature (*T*) and total annual precipitation (P) (step 2 in Fig. 2). Using the Mahalanobis distance aims at rescaling the *T* and *P* axes in order to account for the correlations between the two variables and to calculate standard Euclidean distance in a transformed space having unit variance. In other words, it aims to reduce the dominance of one climatic variable over the other when computing “climatic” distance between years. A trapezoidal probability is then assigned to the *l*-1 remaining years of the reference period, as follows (step 3 in Fig. 2):

(1) P(i)=2(m+1−i)/m(m+1),i=1,…,m

(2) P(i)=0,i=m+1,…,l−1

where *P*(*i*) is the probability assigned to the year with rank *i; i* is the rank of the remaining years of the reference period sorted in order of increasing Mahalanobis distance to the originally selected year; *m* is a number selected randomly at each sub-period selection from the interval [*n*-1, *l*-1]. The year closest to the year originally retained has the highest probability (*P*(1)= 2/*m* + 1) and the farthest years has the lowest probability (*P*(*m*) = 2/*m*(*m* + 1) and *P*(*i*) = 0 for *i* > *m*).

The *n*-1 remaining years of the sub-period are then selected from the *l*-1 remaining years of the reference period according to the trapezoidal probability distribution giving more chance to be selected to the years which are the closest to the initial year according to the Mahalanobis distance defined in the *T* and *P* space (step 4 in Fig. 2). The trapezoidal distribution allows only the *m* years closest to the initial year retained, to be selected in the sub-period. This gives more chance to years with similar climatic conditions to be selected in order to generate more climatically contrasted sub-periods. However, varying randomly *m* for each sub-period generation also allows years with different climatic conditions to be selected. This aims at creating a continuum of climatic conditions, from similar to contrasted, between the sampled sub-periods in view of evaluating the model transferability under increasing climate contrasts. In case the new created sub-period was already sampled, it is not retained (step 5 in Fig. 2). The procedure (steps 1 to 5 in Fig. 2) is repeated until the required number of sub-periods is reached (step 7 in Fig. 2).

The random selection of years in the proposed procedure allows a larger number of sub-periods to be selected than with a deterministic procedure (where the closest years to the originally retained year are selected). In fact, in the best case, the deterministic procedure provides a number of sub-periods equal to the number of observed years (e.g. 30 sub-periods for a 30-year reference period). The number of calibration sub-periods which can be generated by the proposed technique is similar to the random bootstrap SST technique ($C_{n}^{l}$). That is why its application requires a priori selection of the number of permitted calibration exercises. Similarly to the three benchmark SST (See Section "Three benchmark SST techniques"), all *n*-year periods which do not have any year in common with a given *n*-year calibration period can be considered as independent validation exercises with the GDSST.

## MATLAB code of the split sample tests

The routine “SST.m”, presented below and contained in the GDSST package (see supplementary material), allows generating sub-periods by GDSST [5] and by three benchmark Split Sample Tests: (i) sliding-window SST [2]; (ii) random bootstrap SST [1,3]; and (iii) 4-sub-period DSST [4].

The arguments of the routine are:

OptSST: variable used to set the SST to be used. It must be set to 'GSST' for GDSST, 'Mobile' for sliding-window SST, 'Rand_part' for random bootstrap SST, and '4PDSST' for 4-sub-period DSST.

AnnualPrecip: array of *2 x n* dimension. The first column is for years and the second column for annual precipitation. n the number of years of the reference period.

AnnualTemp: array of *2 x n* dimension. The first column is for years and the second column for mean annual temperature. *n* the number of years of the reference period. nsousperiod: number of sub-periods to be generated by GDSST or random bootstrap SST durationSubP: duration of the sub-periods expressed in years.

The routine gives the following outputs: echantillon: a four-column array containing all the independent calibration-validations exercises. Each line contains one calibration-validations exercise. The first column contains the order of calibration period. The second column represents the order of validation period. The third and fourth columns represent the changes in temperature (*ΔT)* and in precipitation (*ΔP*), respectively, between calibration and validation period. The order of subperiod is the same that Combination array.

Combination: contain the years composing the generated sub-periods. Each line contains one subperiod. The order of subperiod in this array is used in echantillon array.

The MATLAB© code of the routine “SST.m”, is presented as follow:







## Application example

The GDSST package (see supplementary material) includes an application example of the four split-sampling methods using the climatic data from five catchments in northern Tunisia (PTfile.mat), it can be run via the Matlab script (MainSST.m).

### The data file: “PTfile.mat”

This matlab file contains the dataset of an application example from five catchments in northern Tunisia (Rhezala, Melah, Maaden, Joumine and El Abid). See Dakhlaoui et al. [5] for more details about the catchments. It contains an array PT of 30 × 11 dimension. The first column of the PT array contains the years, for each catchment two columns are reserved, one for the annual precipitation and the second for mean annual temperature.

The reference period is from 1st September 1970 to 31st August 2000. It was based on the hydrological years (from the 1st of September to the 31st of August).

The PT array of the “PTfile.mat” of the application example of the five catchments of the northern Tunisia, is presented below. The first column contains the years (1971–2000), the second and third columns are reserved respectively to annual precipitation and mean annual temperature of the catchment 1, the fourth and fifth columns are reserved respectively to annual precipitation and mean annual temperature of the catchment 2, etc. The users can implement the new and benchmark split-sample approaches with their own data, by adapting the dimension of this table this to the number of their study catchments and years.

**Table:** 

1971	510,2	17,3	1047,2	15,7	1032,5	15,7	885,0	15,9	911,8	16,1
1972	661,0	16,6	1009,6	14,9	946,5	14,8	743,3	15,0	775,1	15,1
1973	730,6	17,0	1193,1	15,2	1084,3	15,2	897,1	15,4	905,7	15,6
1974	451,2	17,4	636,3	15,7	730,2	15,8	596,6	15,9	620,6	16,1
1975	655,5	16,5	851,6	14,9	1005,5	15,1	743,0	15,3	796,6	15,5
1976	504,0	16,8	819,3	15,0	944,6	15,2	804,5	15,4	805,3	15,6
1977	637,9	17,3	797,8	15,9	933,6	16,2	706,6	16,2	741,6	16,5
1978	355,6	17,0	787,9	16,0	929,0	16,2	741,3	16,0	778,1	16,2
1979	510,1	17,2	791,7	16,7	829,2	16,8	741,9	16,1	728,5	16,4
1980	542,0	16,5	837,9	15,7	1087,0	15,8	933,7	15,4	890,3	15,7
1981	372,3	16,9	843,0	16,2	816,6	16,4	764,1	15,9	718,4	16,2
1982	500,2	18,1	897,5	17,2	973,7	17,3	781,7	16,9	819,8	17,2
1983	562,6	17,6	777,3	16,5	934,7	16,7	848,0	16,4	794,4	16,6
1984	584,9	17,0	991,4	15,8	978,4	16,0	757,3	15,9	805,9	16,1
1985	516,4	17,3	959,3	16,2	1105,1	16,3	850,7	16,2	951,0	16,4
1986	414,8	17,7	718,9	16,2	868,9	16,5	747,3	16,5	768,9	16,8
1987	734,5	17,3	1206,6	16,5	1244,1	16,7	896,6	16,3	959,2	16,6
1988	200,9	18,6	663,7	17,8	712,5	18,0	416,7	17,7	575,8	17,9
1989	354,9	17,9	660,7	16,8	708,9	17,0	587,0	16,7	568,9	16,9
1990	511,4	18,2	553,7	17,1	906,7	17,3	638,7	17,1	634,9	17,3
1991	624,6	17,6	1049,5	16,2	1127,1	16,4	993,4	16,3	1009,0	16,6
1992	656,2	17,3	977,5	15,6	956,0	15,9	768,8	15,7	775,0	16,0
1993	488,1	17,8	648,4	16,4	660,8	16,7	588,2	16,5	605,6	16,8
1994	482,1	18,5	654,9	17,2	754,4	17,5	494,2	17,3	590,4	17,6
1995	303,4	18,4	651,0	16,9	745,5	17,1	537,5	17,1	610,5	17,3
1996	861,2	18,0	1062,8	16,3	1197,7	16,6	989,7	16,5	1200,7	16,7
1997	395,4	18,3	627,9	16,8	721,5	17,1	526,9	17,0	589,9	17,2
1998	588,5	18,4	1123,6	16,8	1258,8	17,1	980,0	17,0	1114,1	17,2
1999	631,5	18,2	1240,8	17,1	941,0	17,4	777,1	17,3	852,1	17,6
2000	569,4	18,7	636,7	17,6	704,0	17,9	512,7	17,8	583,7	18,1

### The main program: “MainSST.m”

The main program allows reading the needed data for the SST “PTfile.m” and running the SST by calling the “SST.m” routine.

Three variables need to be set by the user:

OptSST: Variable that define the SST to be used. It must be set to 'GDSST' for GDSST, 'Mobile' for sliding-window SST,

'Rand_part' for random bootstrap SST, and '4PDSST' for 4-sub-period DSST.

nsousperiod: number of sub-periods to be generated by GDSST or random bootstrap SST durationSubP: Duration of the sub-periods expressed in years.

The code give the following outputs:

Echantillon2: a four column array containing all the independent validations exercises. The first column contain the order of calibration period, the second the order of validation period, the third contain the change in temperature between calibration and validation period *ΔT* and the last contains the relative change in precipitation *ΔP*. Validations exercises from the first catchment are ranged in the first lines, then the second catchment, etc. The order of catchments is the same as PTfile.m

Combination2: contain the years composing the generated sub-periods. Each line contains one sub-period. The first nsousperiod lines contain sub-periods from the first catchment, then the second catchment, etc. The order of catchments is the same as PTfile.m figure.m: Scatter representing the calibration-validation exercises generated by the selected SST expressed in term of *ΔT* and *ΔP*.

The MATLAB© code of the main program “MainSST.m” is presented as follow:



## Implementation details

The MATLAB© codes (MainSST.m and SST.m) and the data file (PTfile.mat) must be put in the same folder. The three variables that need to be set by the user (OptSST, nsousperiod and durationSubP) have to be set directly in the MATLAB© code of the routine (SST.m). The SST could be performed by running “MainSST.m”

## Results of the application example

As an illustration, the three benchmark SST techniques and the proposed GDSST were applied to the application example described above. The techniques were compared as regards to the number of validation exercises and to the precipitation-temperature differences they provided. The length of the sub-periods was set to 8 years for the sliding-window SST, the random bootstrap SST and the GDSST. However, for the 4-sub-period DSST, the 30-year reference period was spread over 7‒8 years hot/dry, hot/wet, cold/dry and cold/wet sub-periods (since 30 is not a multiple of four). The random bootstrap SST results in a large number of possible sub-periods if fully applied to a 30-year period (around six millions 8-year sub-periods). Due to limited time budget for model calibration and validation, we decided to use only 100 randomly selected sub-periods for each catchment. For sake of fair comparison, the same number of randomly selected sub-periods was set with the GDSST. Note that the number of sub-periods for the two other techniques is already limited by their design: 23 sub-periods for the sliding-window SST and four for the 4-sub-period DSST.

**Fig. 3:**
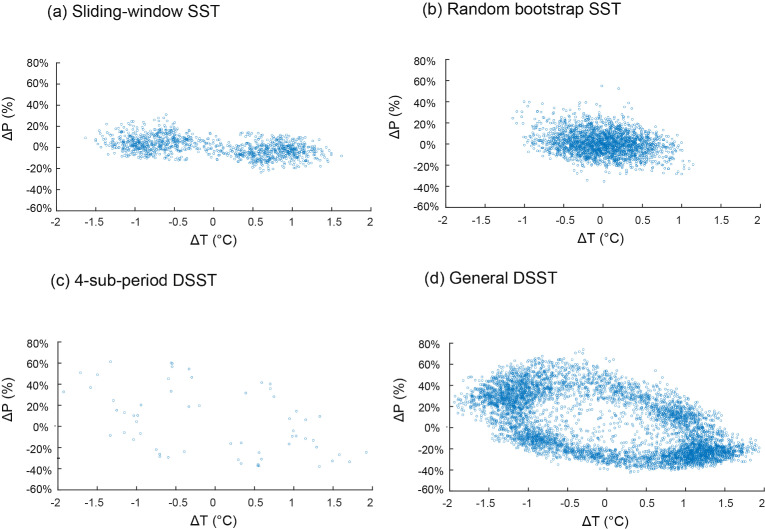
The validation exercises according to four split-sample methods applied over a 30-year reference period (1970‒2000) in the five studied basins: (a) sliding-window SST; (b) random bootstrap SST; (c) 4-sub-period DSST; and (d) the proposed General DSST. *ΔT* and *ΔP* represent respectively the differences in mean annual temperature and the relative difference in annual precipitation between the calibration and validation sub-periods.

Fig. 3 shows the scatter plots generated by the MATLAB© code with the different sampling techniques according to the differences in mean annual temperature and precipitation between the validation and calibration sub-periods (*ΔT* and *ΔP*). The figure allows the spread of the sample provided by each sampling technique to be evaluated in terms of *ΔT* and *ΔP*.

Fig. 3a shows the sample offered by the sliding-window SST technique when applied to the five study catchments. It provided 1 495 possible validation exercises for 115 (23 × 5 basins) calibration exercises. The differences between the different sub-periods in mean precipitation ranged from −20% to +25% and the differences in temperature ranged from −1.8 °C to +1.8 °C. When looking at the random bootstrap SST technique (Fig. 3b), it provided 5800 possible validation exercises for a total of 500 calibration (100 × 5 basins) exercises. The differences between the different sub-periods in mean precipitation ranged from −35% to + 50%, and in temperature from −1.4 °C to +1.4 °C. The 4-sub-period DSST (Fig. 3c) provided 60 possible validation exercises from 20 (4 × 5 basins) calibration sub-periods. The differences in mean precipitation obtained ranged from −40% to +60%, and in temperature from −2 °C to +2 °C. Like the random bootstrap SST, the proposed GDSST (Fig. 3d) provided 9 320 possible validation exercises for a total of 500 calibration (100 × 5 basins) exercises. However, the differences in mean precipitation obtained ranged from −45% to +80%, and in temperature from −2 °C to +2 °C. It should be noted the limited redundancy between the different sampling techniques. In fact sub-periods identified by one approach, are not identified by another. This can be explained by the high number of possible sub-periods (around six million sub-period per catchment) compared the limited number of sampled sub-periods generated by each of the four split-sample methods (4–100 sub-periods per catchment).

Although the sliding-window SST technique provided numerous validation exercises, the differences in *P* (*ΔP*) are less contrasted than those offered by the three other techniques. The sliding-window technique thus appears to depend too much on the historical climate trends to detect extremely contrasted sub-periods for calibration. Using this method, Coron et al. [2] found well contrasted precipitation in southeast Australia. However, the authors reported precipitation trends that contributed to obtain a significant contrast in precipitation characteristics between different periods. In northern Tunisia, continuous sliding periods were unable to provide sufficiently contrasted periods because there was no trend in precipitation during the historical study period, as shown by Dakhlaoui et al. [4]. In addition, the study area presents high inter-annual precipitation variability (see also [4]). Using continuous sub-periods thus smooths the average precipitation in the sub-periods, thereby reducing the climate contrast between them. However, this is not the case for temperature, for which the sliding-window SST technique provided significant differences in *T* (*ΔT*) due to the increasing temperature trends in northern Tunisia over 1970‒2000 [4]. The random bootstrap SST technique provided an important number of validation exercises (5 800). However it led to limited differences in *T* (*ΔT*) and a poor distribution of the sample with high concentration in the center of the figure, where there is the least significant contrast to test model parameter transferability. The 4-sub-period DSST provided more contrasted *ΔT* and *ΔP* than the sliding-window and random bootstrap SST. Indeed it is based on a sampling technique generating highly climate-contrasted sub-periods. However, although it explored contrasted climatic conditions in the historical period, the technique provides very few insights into moderate *ΔT* and *ΔP* compared to the other techniques. The oriented bootstrap of the GDSST provided more validation exercises than the random bootstrap SST, although both techniques were based on the same number of calibration exercises (500). This can be explained by the fact that the oriented bootstrap favours the selection of independent sub-periods by reducing overlap between them. In addition, the GDSST provided a better spread of validation periods. Indeed, contrary to the random bootstrap technique in which the validation exercises were concentrated in the zone of ΔT and ΔP near 0, the sample provided by the GDSST technique was more concentrated at the extremes ΔT and ΔP, which are the most contrasted sub-periods to test the parameter transferability. Hence, the differences in mean precipitation and temperature between the different sub-periods ranged respectively from −45% to +80%, and from −2 °C to +2 °C, thus providing a more marked climatic contrast between the calibration and validation periods compared with the previous techniques (see Fig. 3).

It should be noted that the random bootstrap technique theoretically includes all the spread of ΔT and ΔP provided by the other techniques tested. In other words, the theoretical limits of the tested combinations (if all possible combinations were sampled) should be as large as the largest limits provided by all the other techniques. However, the problem is that the application of a bootstrap on all combinations would require excessive computation time and would lead to a very large number (~6 million of 8-year sub-periods) of combinations that could obviously not be tested through cross-validation with hydrological models. The proposed GDSST has the advantage to be more effective: with only a limited number of calibration exercises (100), it provides a large number of sub-periods from similar to contrasted conditions in terms of precipitation and temperature, while ensuring that the most climatically contrasted sub-periods are sampled.

## Conclusion

We present in this paper the MATLAB© code and an application example of the GDSST proposed by Dakhlaoui et al. [5]. The code allows to user to generate subperiods and corresponding calibration-validation exercises by the new GDSST and three benchmark Split Sample Tests. The code allows the user to visualize the generated subperiods in a scatter plot representing the calibration-validation exercises expressed in term of the differences in mean annual temperature and precipitation between the validation and calibration sub-periods (*ΔT* and *ΔP*). This figure allows evaluating the spread of the sample provided by the sampling technique, before to use it in a DSST exercise. In the provided application example, the GDSST was compared to three other existing techniques to select sub-periods over a 30-year past period on a set of five basins under semi-arid conditions in northern Tunisia. We showed that the GDSST outperformed the other split-sample techniques by providing a large number of sub-periods from similar to contrasted conditions in terms of precipitation and temperature, while ensuring that the most climatically contrasted sub-periods are sampled. This technique thus allows parameter transferability to be tested under wide ranges of climate conditions, which is a key step to assess robustness of hydrological models under past and future climate variability. The users can implement the new and the benchmark split-sample approaches with their own data, by adapting the input file to their data.

## Declaration of Competing Interest

The authors declare that they have no known competing financial interests or personal relationships that could have appeared to influence the work reported in this paper.

## Appendix Supplementary materials

Image, application 1
