# Supplementary material for: A general differential split-sample test to select sub-periods of discontinuous years gathering similar to different climate conditions
Source: MethodsX. 2020 Jul 23;7:101008. doi: 10.1016/j.mex.2020.101008 (PMC7451689; doi:10.1016/j.mex.2020.101008)
Supplement: Supplementary file 1 [file mmc1.zip › GDSST Package User Guide.pdf]

Package ‘GDSST’

August 4th, 2020

Version : 1.0

Title : General Differential Split Sample Test

Authors : Hamouda DAKHLAOUI, Denis RUELLAND, Yves TRAMBLAY

Maintainer : Hamouda DAKHLAOUI <hammouda.dakhlaoui@laposte.net>

Description : A Matlab script (SST.m) that Generate sub-periods by General Differential Split Sample Test (Dakhlaoui et al., 2019) and three benchmark Split Sample Test : (i) sliding-window SST (Coron et al., 2012); (ii) random bootstrap SST (Coron, 2013); and (iii) 4-sub-period DSST (Dakhlaoui et al., 2017). An application example of the four SST methods using the climatic data from 5 catchment in northern Tunisia (PTfile.mat) can be run via the Matlab script (MainSST.m)

Date/Publication : 2019-05-10 18:21:30 UTC

References

Coron, L., Andréassian, V., Perrin, C., Lerat, J., Vaze, J., Bourqui, M., Hendrickx, F., 2012. Crash testing hydrological models in contrasted climate conditions: an experiment on 216 Australian catchments. Water Resour. Res., 48, W05552. doi :10.1029/2011WR011721

Coron, L., 2013. Les modèles hydrologiques conceptuels sont-ils robustes face à un climat en évolution? ISIVE, AgroParisTech, 364 p.

Dakhlaoui, H., Ruelland, D., Tramblay, Y., Bargaoui, Z., 2017. Evaluating robustness of conceptual rainfall-runoff models under climate variability in northern Tunisia. J. Hydrol., 550, 201–217. doi:10.1016/j.jhydrol.2017.04.032

Dakhlaoui, H., Ruelland, D., and Tramblay Y. (2019). A bootstrap-based differential split-sample test to assess the transferability of conceptual rainfall-runoff models under past and future climate variability. Journal of Hydrology. https://doi.org/10.1016/j.jhydrol.2019.05.056

Matlab topics documented:

SST.m 2

PTfile.mat 3

MainSST.m 4

SST.m

Description

Matlab script that generate sub-periods by General Differential Split Sample Test (Dakhlaoui et al., 2019) and three benchmark Split Sample Test : (i) sliding-window SST (Coron et al., 2012); (ii) random bootstrap SST (Coron, 2013); and (iii) 4-sub-period DSST (Dakhlaoui et al., 2017).

Usage

```
[echantillon, Combination] = SST (OptSST, AnnualPrecip,
AnnualTemp, nsousperiod, durationSubP)
```

Arguments

`OptSST`	:	variable used to set the SST to be used. It must be set to 'GSST' for GDSST, 'Mobile' for sliding-window SST, 'Rand_part' for random bootstrap SST, and '4PDSST' for 4-sub-period DSST.
`AnnualPrecip`	:	array of *2 x n* dimension. The first column is for years and the second column for annual precipitation. *n* the number of years of the reference period.
`AnnualTemp`	:	array of *2 x n* dimension. The first column is for years and the second column for mean annual temperature. *n* the number of years of the reference period.
`nsousperiod`	:	number of sub-periods to be generated by GDSST or random bootstrap SST
`durationSubP`	:	duration of the sub-periods expressed in years.

Outputs

`echantillon`	:	a four column array containing all the independent calibration-validations exercises. Each line contain one calibration-validations exercise. The first column contain the order of calibration period , the second the order of validation period, the third contains the changes in temperature between calibration and validation period ΔT and the last contains the changes in precipitation ΔP. The order of subperiod is the same that `Combination` array.
`Combination`	:	contain the years composing the generated sub-periods. Each line contain one subperiod. The order of subperiod in this arry is used in `echantillon` array.

PTfile.mat

Description

Contain the dataset of an application example from 5 catchment in northern Tunisia (Rhezala, Melah, Maaden, Joumine and El Abid). See Dakhlaoui et al. 2019 for more details about catchments. It contains an array PT of *30x11* dimension. The first column of the PT array contain the years, for each catchment two column are reserved, one for the annual precipitation and the second for mean annual temperature.
The reference period is from 1st September 1970 to 31st August 2000.

MainSST.m

Description

Main program allowing to run the application example from the 5 catchment in northern Tunisia (PTfile.m) .

Variable to be set

`OptSST`	:	variable used to select the SST to be used. It must be set to 'GSST' for GDSST, 'Mobile' for sliding-window SST, 'Rand_part' for random bootstrap SST, and '4PDSST' for 4-sub-period DSST.
`nsousperiod`	:	number of sub-periods to be generated by GDSST or random bootstrap SST
`durationSubP`	:	duration of the sub-periods expressed in years.

Outputs

`Echantillon2`	:	a four column array containing all the independent validations exercises. The first column contain the order of calibration period, the second the order of validation period, the third contain the change in temperature between calibration and validation period ΔT and the last contains the relative change in precipitation ΔP. Validations exercises from the first catchment are ranged in the first lines, then the second catchment, etc. The order of catchments is the same as `PTfile.m`
`Combination2`	:	contain the years composing the generated sub-periods. Each line contain one sub-period. The first `nsousperiod` lines contain sub-periods from the first catchment, then the second catchment, etc. The order of catchments is the same as `PTfile.m`
`figure.m`	:	Scatter representing the calibration-validation exercises generated by the selected SST expressed in term of ΔT and ΔP.
